# Comparative Transcriptome Analysis Reveals Key Pathways and Hub Genes in Rapeseed During the Early Stage of *Plasmodiophora brassicae* Infection

**DOI:** 10.3389/fgene.2019.01275

**Published:** 2020-01-17

**Authors:** Lixia Li, Ying Long, Hao Li, Xiaoming Wu

**Affiliations:** Key Laboratory of Biology and Genetic Improvement of Oil Crops, Ministry of Agriculture and Rural Affairs, Oil Crop Research Institute, Chinese Academy of Agricultural Sciences, Hubei, China

**Keywords:** *Brassica napus*, *plasmodiophora brassicae*, transcriptome, hub genes, glucosinolate, plant hormone

## Abstract

Rapeseed (*Brassica napus* L., AACC, 2n = 38) is one of the most important oil crops around the world. With intensified rapeseed cultivation, the incidence and severity of clubroot infected by *Plasmodiophora brassicae* Wor. (*P. brassicae*) has increased very fast, which seriously impedes the development of rapeseed industry. Therefore, it is very important and timely to investigate the mechanisms and genes regulating clubroot resistance (CR) in rapeseed. In this study, comparative transcriptome analysis was carried out on two rapeseed accessions of R- (resistant) and S- (susceptible) line. Three thousand one hundred seventy-one and 714 differentially expressed genes (DEGs) were detected in the R- and S-line compared with the control groups, respectively. The results indicated that the CR difference between the R- and S-line had already shown during the early stage of *P. brassicae* infection and the change of gene expression pattern of R-line exhibited a more intense defensive response than that of S-line. Moreover, Kyoto Encyclopedia of Genes and Genomes (KEGG) analysis of 2,163 relative-DEGs, identified between the R- and S-line, revealed that genes participated in plant hormone signal transduction, fatty acid metabolism, and glucosinolate biosynthesis were involved in regulation of CR. Further, 12 hub genes were identified from all relative-DEGs with the help of weighted gene co-expression network analysis. Haplotype analysis indicated that the natural variations in the coding regions of some hub genes also made contributed to CR. This study not only provides valuable information for CR molecular mechanisms, but also has applied implications for CR breeding in rapeseed.

## Introduction

*Plasmodiophora brassicae* Wor. (*P. brassicae*), an obligate and biotrophic pathogen of Rhizaria (Schwelm et al., 2015), could infect over 3,700 species in Brassicaceae (Hwang et al., 2012), and lead clubroot which has caused significant economic losses every year (Dixon, 2009). The *P. brassicae* has been discovered in more than 60 countries or regions (Dixon, 2009), the life cycle of which consists of dormant stage of resting spores, germination stage of resting spores, and secondary zoospore reinfection. Once the conditions are suitable, primary zoospores are released from the resting spores to infect the root hairs when feel the stimulation of relevant signaling molecules secreted by host plants (Aist and Williams, 1971; Kageyama and Asano, 2009; Rolfe et al., 2016). The primary plasma mass is formed in the root hairs, and then divided to form secondary sporangium, from which the secondary zoospores are released. Secondary zoospores directly infect cortical cells, where secondary plasma mass form. Finally, the secondary plasma mass is divided to form mature resting spores, which are scattered in the soil and become the initial infection source in the coming year (McDonald et al., 2014). The resting spores of *P. brassicae* can survive for at least 7 years in the soil (Karling, 1968). Once contaminated, the field will no longer suitable for Brassicaceae crops (Howard et al., 2010). As early as 1930, pathogenic specialization has been found in *P. brassicae* (Honig, 1931). There are great differences in the biological and molecular characteristics of different pathogenic strains, which impede the research progress of pathogenesis. Up to now, only a few genes considering as the pathogenic factors in *P. brassicae* have been identified (Ando et al., 2006; Bulman et al., 2006; Feng et al., 2010).

Plants could defense pathogens with the help of physical (such as cell wall, cuticle, waxy layer, and xylogen) or chemical barriers (such as phenols, saponins, and mustard oil). Once the above defense is breached, the plant activates its defense immune system immediately, which consists of pathogen-associated molecular pattern (PAMP)-triggered immunity (PTI) and effector-triggered immunity (ETI, Jones and Dangl, 2006). PTI is the basal immune response, which could be inhibited by the effectors secreted by pathogens. Then, effector could be recognized by R protein in plants, consequently triggering a more dramatic immune response ETI (Bent and Mackey, 2007). Most R proteins were reported containing conserved motifs such as toll-interleukin receptor (TIR), nucleotide-binding (NB), leucine-rich repeat (LRR), coiled-coil (CC), or leucine zipper (Liu et al., 2007). Despite many clubroot resistance (CR) sites were identified in Brassicaceae, only three of which, *CRa* (Ueno et al., 2012), *Crr1a* (Hatakeyama et al., 2013), and *CRb* (Hatakeyama et al., 2017), have been cloned and found containing TIR-NB-LRR or NB-LRR.

In recent years, many studies were focused on the molecular mechanisms of CR with the help of “-omics” approach, especially in *Arabidopsis thaliana* and *Brassica* species. In *Arabidopsis*, the expression level of genes related to growth, sugar-phosphate metabolism, defense, and plant hormone had undergone large-scale changes after inoculation (Siemens et al., 2006). Some studies also elucidated the role of genes associated with metabolism, hormonal signaling pathways, and stress response (Jubault et al., 2008; Ludwig-Muller et al., 2009; Agarwal et al., 2011; Schuller et al., 2014). In *Brassica*, some studies indicated that the genes involved in the signaling metabolism of jasmonate and ethylene, defensive deposition of callose, and the biosynthesis of indole-containing compounds were all significantly up-regulated in clubroot-resistant plants compared with susceptible cultivars (Chu et al., 2014). It was confirmed that genes associated with PAMPs, calcium ion influx, hormone signaling, pathogenesis related, and cell-wall modification played important roles in the interactions between *Brassica rapa* and *P. brassicae* (Chen et al., 2015). More recently, proteomic analysis found that two proteins related to salicylic acid (SA) mediated systemic acquired resistance and two proteins related to jasmonic acid (JA)/ethylene (ET) mediated induced systemic resistance in Chinese cabbage (Ji et al., 2018). Compared with the susceptible accessions, the genes involved in cell wall, SA signal transduction, phytoalexin synthesis, chitinase synthesis, Ca^2+^ signaling, and reactive oxygen species were significantly activated in resistant cabbage (Devos et al., 2006; Zhang et al., 2016). Remarkably, a series of studies were conducted to explore the relationship of glucosinolate (GLS) and CR in different species, which could provide more valuable information on CR mechanisms. GLS had been proved associated with clubroot disease symptoms both in *Arabidopsis* and *Brassica* species (LudwigMuller et al., 1997; Ludwig-Muller et al., 1999a; Ludwig-Muller et al., 1999b; Ludwig-Muller et al., 2009).

Rapeseed (*Brassica napus* L., AACC, 2n = 38) is one of the most important *Brassica* crops around the world, which provides not only edible oil for human, but also protein-rich feed for animals. With intensified rapeseed cultivation, the incidence and severity of clubroot has also increased, which impedes the development of the rapeseed industry seriously. Up to now, large number of studies are focused on screening of resistant materials or mapping of C*R* genes/quantitative trait loci (Manzanares-Dauleux et al., 2000; Tewari et al., 2005; Werner et al., 2008; Zhang et al., 2015), but the resistance mechanisms of rapeseed against clubroot is still not clear. The results of protein level changes on infected *B. napus* showed that there were differences in proteins related to lignin synthesis, cytokinins metabolism, glycolysis, intracellular calcium ion balance, and reactive oxygen species detoxification (Cao et al., 2008). MicroRNA (miRNA) analysis on infected *B. napus* root showed that differential expressed genes (DEGs) of miRNA targets were predicted involved in transcriptional factors activity, hormone, and plant defense response (Verma et al., 2014). Furthermore, it was pointed out that genes related to the IAA (indole-3-acetic acid), SA, and JA pathways were involved in the reaction of *B. napus* to *P. brassicae* (Xu et al., 2016; Prerostova et al., 2018). The latest research showed that phenylpropanoid pathway was instrumental in resistance to clubroot disease progression in resistant line (Irani et al., 2019).

In this study, we investigated the early defense response of different resistant level rapeseed accessions to *P. brassicae*. Comparative dynamic analysis of the number of DEGs in R- (resistant) and S- (susceptible) line suggested that the differences between the R- and S-line had already shown in the early stage and the R-line was more sensitive to the invasion of *P. brassicae*. Functional enrichment analysis of DEGs revealed the important pathways responsible for CR in rapeseed. Based on that, the hub genes in each important pathway were screened out combining with the weighted gene co-expression network analysis (WGCNA). Haplotype analysis indicated that the natural variations in the coding regions of some hub genes made contributed to CR. This study not only provided valuable information for CR molecular mechanisms, but also had applied implications for CR breeding in rapeseed.

## Materials and Methods

### Plant Materials, Resistance Identification, and Sampling

Two rapeseed accessions of 28,669 (resistant, R-line; *B. napus*, 2n = 4x = 38) and YJ-8 (susceptible, S-line; *B. napus*, 2n = 4x = 38) with contrasting performance on resistance to *P. brassicae*, which have experienced several resistance identifications in different environments, were used in the present study. These two lines were semi-winter type rapeseed, which were collected from the National Mid-term Gene Bank for Oil Crops of China. The R-line was double-high oil quality with GLS (94.5 µmol/g) and erucic (24.5%), while the S-line was double-low oil quality with GLS (28.5 µmol/g) and erucic (0%). The pathogen used in this study was collected from the infected field (IF) of Dangyang, China, where the pathogen was reported as pathotype 4 based on Williams classification (Ren et al., 2012). The seeds of R- and S-line were germinated on wet filter paper for 7 days, then transferred into the plastic pots filled with 10 L Hoagland nutrient solution adding the Ca(NO_3_)_2_ with 0.945 gram (g) per L additionally for 1 month under a 16 hpi (h) photoperiod at 25°C. Then, the seedlings were transferred into the fermentative soil as the proportion of 10^6^ resting spores per g dry soil with the same condition of the culture room. The methods of making *P. brassicae* suspension and fermentative soil were also as the previous study (Li et al., 2016). We choose the 12, 24, 60, and 96 h post-inoculation of *P. brassicae* as the sampling time points for RNA-seq based on the results of the study (Dobson and Gabrielson, 1983). Three biological replicates of each treatment with one mock-control were performed. The roots of 10 plants were sampled at each replication for RNA sequencing. To verify successful infection, 20 plants of each accession, which were transferred into the fermentative soil with *P. brassicae*, were remained for resistance identification until the 42 days post-inoculation. The evaluation of severity of disease was as reported before (Kuginuki et al., 1999).

### RNA Extraction and Construction of cDNA Sequencing Library

Total RNAs of 32 samples were extracted using the TRIzol reagent (Life Technologies, Carlsbad, California). The RNA quality (degradation and DNA contamination) was monitored on 1% agarose gels electrophoresis. The RNA purity and concentration were checked using the NanoPhotometer^®^ spectrophotometer (IMPLEN, CA, USA), and Qubit^®^ RNA Assay Kit in Qubit^®^ 2.0 Fluorometer (Life Technologies, CA, USA), respectively. The integrity of RNA was assessed using the RNA Nano 6000 Assay Kit of the Bioanalyzer 2100 system (Agilent Technologies, CA, USA). A total amount of 3 µg RNA per sample was used for library preparation using NEBNext^®^ Ultra*™* RNA Library Prep Kit for Illumina^®^ (NEB, USA). In order to select complementary DNA (cDNA) fragments of preferentially 150–200 bp in length, the library fragments were purified with AMPure XP system (Beckman Coulter, Beverly, USA). Then 3 µl USER Enzyme (NEB, USA) was used with size-selected, adaptor-ligated cDNA at 37°C for 15 min followed by 5 min at 95°C before PCR. Then PCR was performed and the products were purified (AMPure XP system). The library quality was assessed using the Agilent Bioanalyzer 2100 system.

### RNA Sequencing and Data Preprocessing

All the 32 libraries were sequenced on the Illumina HiSeq platform and 150 bp paired-end raw reads were generated. Before assemblies, various quality-controlling measures for raw data were conducted. High-quality clean data was obtained by removing reads containing adapter, reads containing ploy-N, and low-quality reads from raw data. The high-quality paired-end clean reads were aligned to the *B. napus* reference genome (*Darmor-bzh*, Chalhoub et al., 2014) using TopHatv2.0.12 (Trapnell et al., 2009). Only uniquely mapped reads were considered for further analyses. The reads number mapped to each gene was counted using HTSeq v0.6.1. Then, the fragment per kilobases of transcript per million reads (FPKM) of each gene was calculated based on the length of the gene and reads count (Trapnell et al., 2010). The software of Microsoft Excel 2010 was used to calculate the person correlation of biological replicates.

### Identification of Differentially Expressed Genes and Real Time-PCR Verification

Differential expression analysis of two samples was performed using the DESeq R package (1.18.0). Genes with an adjusted *p*-value (padj) < 0.05 and |log_2_ (fold change)| > 0 found by DESeq were considered as DEGs. To distinguish the DEGs more clearly, the concept of relative differentially expressed genes (RDEGs) was introduced in this study. A gene was defined as a RDEG, when it was not only identified as DEG between R- and S-line, but also identified as DEG in R-line (compared with the R-mock) or in S-line (compared with the S-mock) at the corresponding time points. RT-PCR was carried out to confirm the RNA-seq results. cDNAs were synthesized from the same RNAs as for RNA-seq. The results analysis performed on LightCycler 480 SYBR Green I Mastermix, and a LightCycler 480II real-time PCR system (Roche, Switzerland). The transcript abundance calculated from three biological and three technical replicates with *Bna.ACTIN7* (*BnaA03g55890D*) as internal control. The fold change was estimated using the 2^−ΔΔCT^ (Livak and Schmittgen, 2001). The gene-specific primers sequences were list (**Table S1**).

### Gene Ontology and Kyoto Encyclopedia of Genes and Genomes Enrichment Analysis of Differentially Expressed Genes

Gene Ontology (GO) enrichment analysis was implemented by the GOseq R package, in which gene length bias was corrected. GO terms with padj ≤ 0.05 were considered as significantly enriched. Kyoto Encyclopedia of Genes and Genomes (KEGG) was carried out online (http://www.genome.jp/kegg/). KOBAS software was used to test the statistical enrichment of differential expression genes in KEGG pathways (Mao et al., 2005). The pathways with padj ≤ 0.01 were considered as significantly enriched. The heat maps performed on the software of Genesis.

### Co-Expression Network Analysis and Prediction of Hub-Genes

The co-expression network analysis was conducted using WGCNA version 1.61 package in R software (Langfelder and Horvath, 2008). Module identification was implemented after merging of modules whose expression profiles were similar with a merge CutHeight of 0.25. The interaction network of hub-genes in module was visualized using Cytoscape 3.5.1.

### Haplotype Analysis

The primers were designed for PCR of three hub genes genomic sequences in a population including 130 accessions (**Table S2**). Single-nucleotide polymorphism (SNP) information of hub genes in the population was obtained by blasting the sequences of the PCR products. The software of Haploview was used to analyze the haplotype in the population. The phenotype data of 130 rapeseed accessions used for the haplotype analysis were obtained by artificial inoculation at seedling stage in greenhouse from Li et al. (2016). The significance of phenotype difference among different haplotypes was detected by *t*-test using Microsoft EXCEL 2010 and visualized by the violin plot (http://shiny.chemgrid.org/boxplotr/).

## Results

### Phenotype Characterization of Two Rapeseed Accessions With Contrasting Resistance to Clubroot in Different Environments

**Figure 1:**
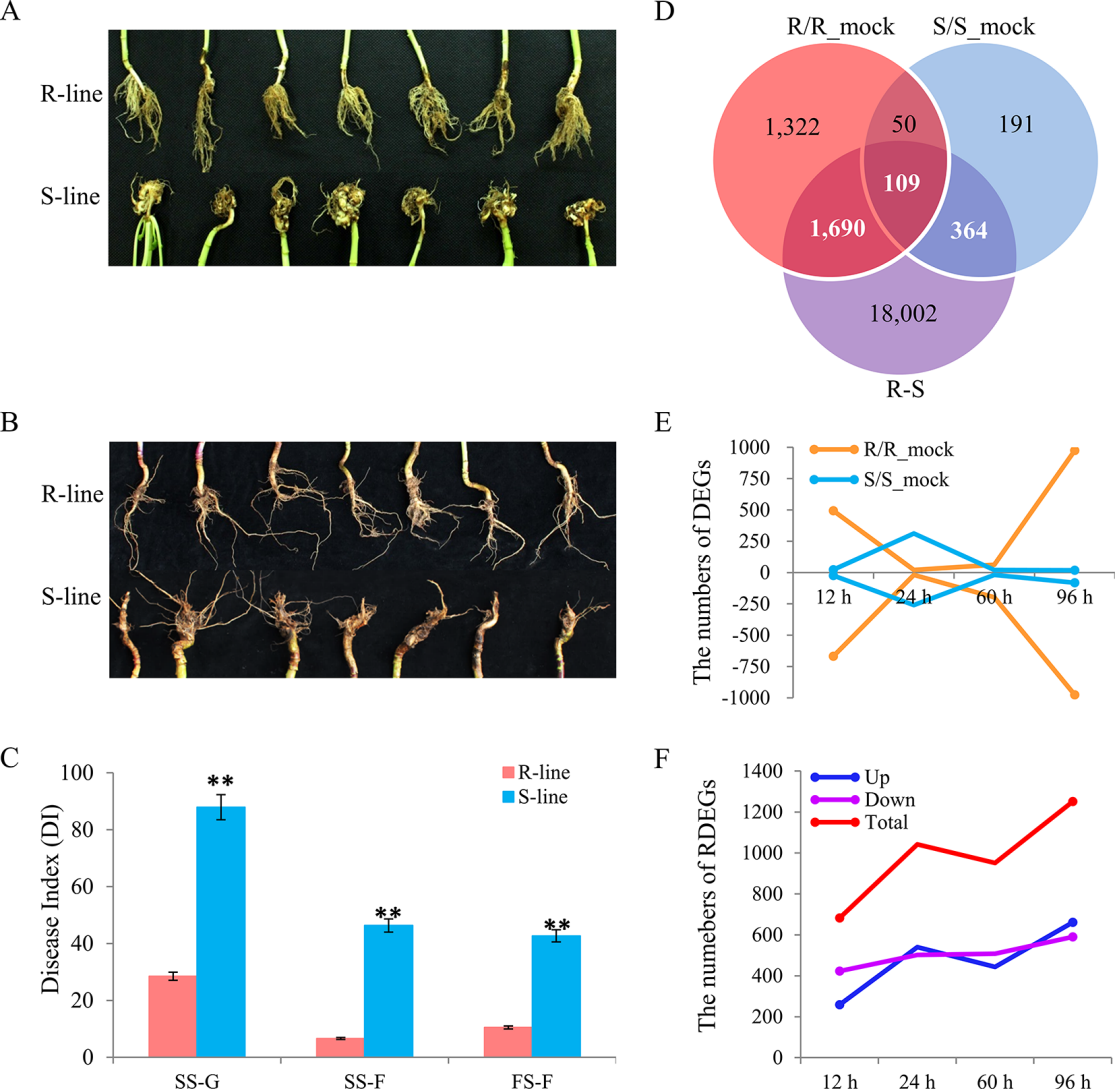
Phenotype characterization in different environments, and identification of differentially expressed genes (DEGs) and relative differentially expressed genes (RDEGs) after infected by *Plasmodiophora brassicae*. Phenotype difference between the R- and S-line at seedling stage **(A)** and flowering stage **(B)**. **(C)** Disease index of the two accessions in different environments. SS-G, SS-F, and FS-F indicated the seedling stage in greenhouse, field, and the flowering stage in field, respectively. **(D)** Venn diagram of DEGs in the R/R_mock, S/S_mock, and R/S. The sum of white and bold numeral represented the number of RDEGs. **(E)** Dynamic variation of up- and down-DEGs in each accession. The negative indicated the number of down-DEGs. The yellow and blue line indicated the DEGs of R- and S-line compared with the corresponding mock group, respectively. **(F)** Dynamic variation of RDEGs. The blue, purple, and red line indicated the up-, down-, and total RDEGs, respectively.

Two rapeseed accessions, R- and S-line (the most constant accessions), were screened out from a natural population containing 472 accessions (Li et al., 2014), which were performed the CR evaluation in three environments (data were not shown on). The morphological differences between R- and S-line in greenhouse by artificial inoculation were showed in **Figure 1A** and that in IF by natural infection were showed in **Figure 1B**. The difference of disease index (DI) between R- and S-line was significant in any of environments (**Figure 1C**). The DI of R-line was 28.5 at the seedling stage in greenhouse by artificial inoculation, while the DI of S-line was 87.91; the DI of R-line was 6.64 at the seedling stage in IF, which was significant lower than that of S-line (46.35). At the flowering stage in IF, the DI of R-line was 10.54, while the S-line was 42.7. It was obvious that artificial inoculation in greenhouse can increase the DI greatly compared with natural infection in field. The result suggested that the resistance difference between these two accessions was highly stable, which were reliable for the further study.

### RNA-Sequencing Analysis and Global Comparison of Transcriptomes on Infected Roots by *Plasmodiophora brassicae* Revealed the Difference of the Early Stages Between R- and S-Line

To explore the molecular basis difference of early defense response induced by *P. brassicae* on different resistant level rapeseed accessions, RNA-seq analysis was conducted to generate transcriptome profiles. RNA was extracted from the roots of the R- and S-line at 12, 24, 60, and 96 h after infected by *P. brassicae* with three biological replicates per treatment, respectively. A total of 32 libraries were constructed and analyzed. In total, approximately 1.77 billion raw reads were generated from the 32 samples, and 1.74 billion high-quality clean reads with an average of 54.32 million clean reads (a total of 8.15G) for each sample were obtained after removing low-quality reads. The GC content of the sequence data from the 32 libraries were all around 46.6%, and the Q30 values were all above 90%, indicating that the quality and accuracy of sequencing data was sufficient for further analyses. On average, 88.9% clean reads were mapped on the *B. napus* reference genome, and about 95.7% of which were matched uniquely (**Table S3**). Pearson correlation coefficients of three biological replicates in each treatment for both the R- and S-lines were high (*R^2^* > 0.90 in most cases, **Figure S1**), which indicated that the RNA-seq data was of high quality and consistency. The number of transcript of each sample was showed after removing the genes with a FPKM value (average of three biological replicates) < 1. In total, 55,240 and 54,538 transcripts were identified in mock-inoculated and inoculated samples by *P. brassicae* of R-line, respectively. Similarly, 55,821 and 55,560 transcripts were detected in S-line, respectively (**Table 1**). Overall, the numbers of expressed genes in any samples accounted for 47.5–50.5% of the 101,040 *B. napus* annotated genes. There was no significant difference in the number of transcript among the different sampling point both in R- and S-line. Similarly, the clean reads also mapped to the *P. brassicae* genome for analysis of the genome enrichment in rapeseed roots. However, it was failed to acquired enough reads to perform the further analysis maybe because that the pathogen invasion time is too short (the longest time-point was 96 h) to get *P. brassicae* genome information in rapeseed roots.

**Table 1:** Transcripts statistics of different sampling point expressed in mock-inoculated and inoculated samples.

	12 h		24 h		60 h		96 h		Total	
	Mock inoculated	Inoculated	Mock inoculated	Inoculated	Mock inoculated	Inoculated	Mock inoculated	Inoculated	Mock inoculated	Inoculated
**R-line**	48,150	49,839	50,280	50,592	49,981	50,880	49,582	50,630	55,240	54,538
**S-line**	48,015	50,104	50,238	50,798	49,869	50,605	51,003	50,868	55,821	55,560

### Identification of Differentially Expressed Genes and Validation of RNA-Sequencing by RT-PCR

Compared with the corresponding mock group, 3,171 and 714 DEGs were detected in R- and S-line with padj) < 0.05, and |log_2_ (fold change)| > 0, respectively. A total of 159 genes were detected in both lines, while 3,012 and 555 genes were R-line specific and S-line specific, respectively (**Figure 1D**). In R-line, 240 DEGs were present at more than one time points (**Figure S2A**). Similarly, 42 DEGs presented at more than one points were identified in S-line (**Figure S2B**). Subsequently, the expression patterns of DEGs identified at different sampling time points in both lines were investigated. At 12 h after inoculated, 493 genes were up-regulated and 668 genes were down-regulated in R-line, while only 23 genes were up-regulated and 24 genes were down-regulated in S-line, respectively. The similar situation was also present at 96 h, 974 genes were up-regulated, and 976 genes were down-regulated in R-line, while only 19 genes were up-regulated and 81 genes were down-regulated in S-line, respectively (**Figure 1E**). The above results indicated that the difference between R- and S-line had already shown in the early stage after being attacked by *P. brassicae*. Overall, the change of gene expression pattern of R-line exhibited a more intense defensive response than that of S-line, which manifested the R-line was more sensitive to the invasion of *P. brassicae*. To explore the key important genes responsible for the difference of CR between the two accessions, 4,567, 10,065, 7,453, and 9,477 DEGs were identified between R- and S-line at 12, 24, 60, and 96 h after inoculated, respectively. Finally, a total of 2,163 RDEGs were screened out from all DEGs for further analyses (**Figure 1D**). In detail, compared with S-line, 259, 540, 443, and 661 up-regulated RDEGs were identified in R-line at 12, 24, 60, and 96 h after inoculation, respectively. Similarly, 423, 502, 508, and 590 down-regulated RDEGs were identified in that of S-line (**Figure 1F**).

To validate the quality of RNA-seq data and difference expressional level, 29 RDEGs were selected randomly for RT-PCR. The relative expression level measured by RT-PCR was converted to fold changes (R/S). All RT-PCR data was collected from three technical replicates for each sampling time point and the strong correlation between the RNA-seq and RT-PCR data were present (*R^2^* = 0.852-0.986, **Figure S3**), which indicated that the transcriptomic profiling data was of reliability.

### Functional Enrichment Analyses of Differentially Expressed Genes and Relative Differentially Expressed Genes

**Figure 2:**
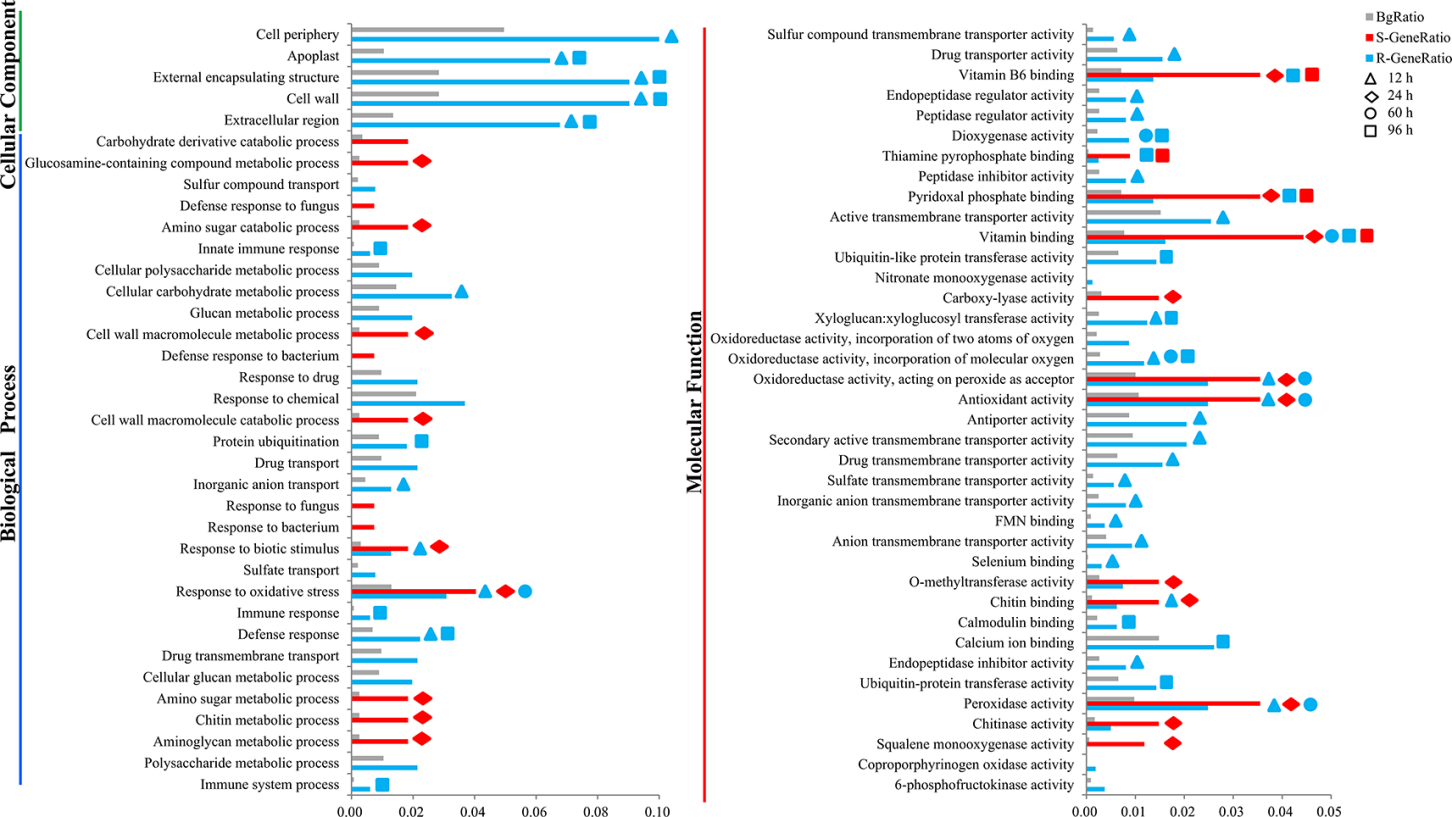
Gene ontology (GO) enrichment analysis of the differentially expressed genes in the R- and S-line. The y-axis meant the GO terms enriched in the R- or S-line belonging to three categories, and the x-axis meant the percentage, which calculated by the formula that the gene number enriched in each term dividing that in corresponding categories. The gray, red, and blue pillars meant the percentage in the background, S-line, and R-line, respectively. The pillars marked with the shape of triangle, rhombus, circle, and square represented the terms enriched at 12, 24, 60, and 96 h after infection, respectively.

To understand the biological mechanisms of CR deeply, GO enrichment analyses of the DEGs in R-line (compared with the R-mock) and S-line (compared with the S-mock) were conducted, respectively. In total, the 3,171 DEGs in R-line were assigned to 60 terms belonging to three categories: biological process (19 terms), cell components (5 terms), and molecular function (36 terms) significantly. Seven hundred fourteen DEGs in S-line were assigned to 26 terms belonging to two categories: biological process (14 terms) and molecular function (12 terms) (**Figure 2**). It was noticeable that 48 terms were enriched in R-line specifically, which were mainly related to immune system process, sulfate transport, extracellular region, cell wall, calcium ion binding, xyloglucosyl transferase activity, and others. While, only 14 terms were enriched in S-line specifically, which were involved in the amino sugar and chitin metabolism (**Figure 2**). In addition, GO analysis was conducted for the DEGs at each stage in both lines. The results showed that 30 pathways were enriched at 12 h after inoculation, accounting for 50% of all pathways in R-line. However, the DEGs at 12 h after inoculation in S-line were not enriched into any pathway. On the contrary, 20 pathways were enriched at 24 h after inoculation, accounting for 76.9% of all pathways in S-line. Compared with the R-line, the S-line showed a delay in response to pathogen invasion, which was mainly reflected in the response to biological stimulation and oxidative stress (**Figure 2**).

**Figure 3:**
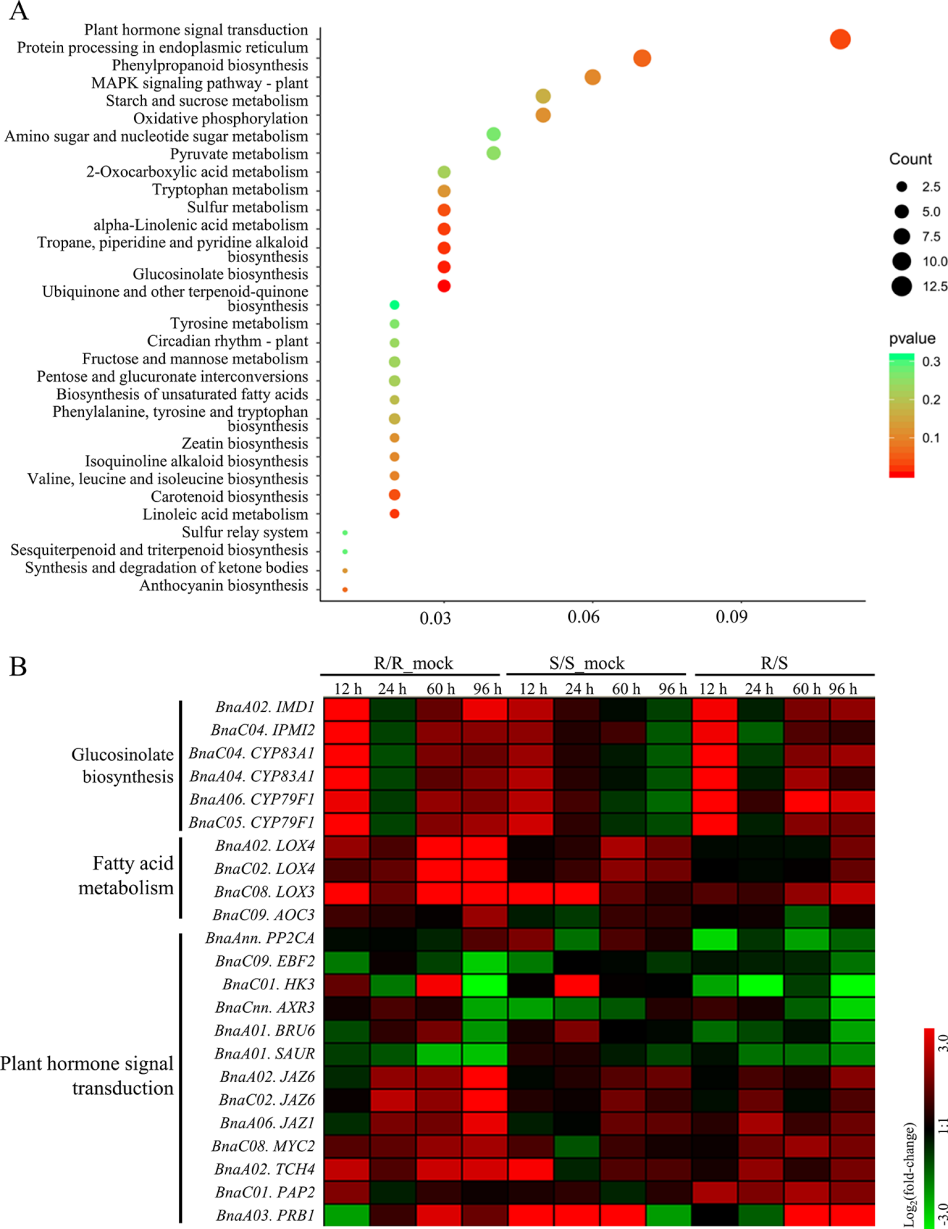
Kyoto Encyclopedia of Genes and Genomes enrichment analysis of relative differentially expressed genes and expressional differential analysis of genes enriched in important pathways. **(A)** The size of dot indicated the number of gene enriched in each pathway, and the color of dot meant the significance (*p* value) of each pathway. **(B)** Heat-maps showed the log_2_ fold-change of the genes enriched in important pathways at each sampling time point after infection.

Furthermore, KEGG analysis was performed on the RDEGs and 30 pathways were showed, only eight of which were enriched significantly with a cutoff value of *p*_value < 0.05 (**Figure 3A**). The results revealed that the pathways of GLS biosynthesis, pyridine alkaloid biosynthesis, fatty acid metabolism, plant hormone signal transduction, sulfur metabolism, tryptophan metabolism, and carotenoid biosynthesis, might involve in the regulation of CR in *B. napus*. In order to show the expression differences of the RDEGs enriched in above pathways, heat map was prepared. It is noticeable that the genes involved in GLS biosynthesis were up-regulation both in R- and S-line at 12 h after inoculation, which were also up-regulation in R-line compared with the S-line. At 96 h after inoculation, the genes involved in GLS biosynthesis were up-regulation in R-line, while down-regulation in S-line. In addition, the expression differences of genes enriched in fatty acid metabolism performed consistently at 96 h after inoculation, which were all up-regulation. In the pathway of plant hormone signal transduction, the genes encoding auxin-responsive protein family were down-regulation in R-line compared with that in S-line. On the contrary, the genes encoding jasmonate-zim-domain protein were up-regulation in R-line compared with that in S-line (**Figure 3B**).

### Construction of Gene Co-Expression Networks and Prediction of Hub Genes Related to Clubroot Resistance in *Brassica napus*

**Figure 4:**
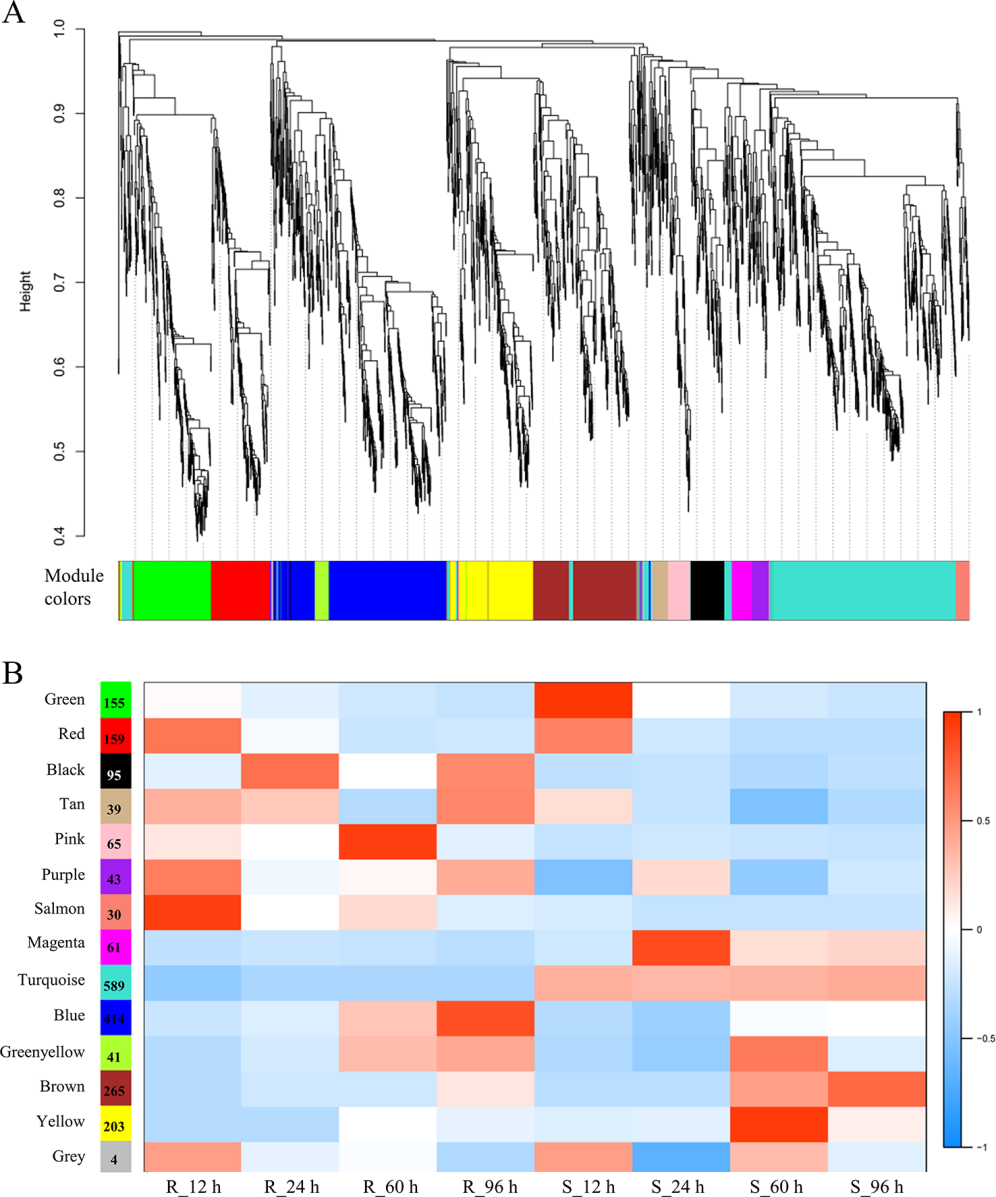
Weighted gene co-expression network analysis of relative differentially expressed genes. **(A)** Hierarchical cluster dendrogram showed co-expression modules. Each leaf (short vertical line) in the tree represented one gene. The genes were clustered based on dissimilarity measure. The major tree branches, corresponded with the color rows below the dendrogram, constituted the modules. **(B)** Module-sample association analysis. Each row corresponded to a module, and each column corresponded to a sample. The number of gene in each module was displayed on the left of each row.

To obtain an insight for understanding the molecular mechanisms in depth and comprehensively, we carried the WGCNA analysis to construct the gene co-expression network. All 2,163 RDEGs were assigned into 13 distinct modules labeled with different colors (**Figure 4A**), except 4 of which cannot be assigned into any module were put into gray module. The module was a cluster of highly interconnected genes with similar expression changes in a physiological process. The number of RDEGs that the modules harbored was varying from 30 (salmon) to 589 (turquoise). Then, we associated the modules with each of samples, which demonstrated that six modules (green, pink, salmon, magenta, blue, and yellow) showed highly correlation with R-line or S-line (**Figure 4B**). Noticeably, the purple module performed opposite expression patterns between R and S-line at any sampling time point and the same simulation happened on turquoise module.

**Figure 5:**
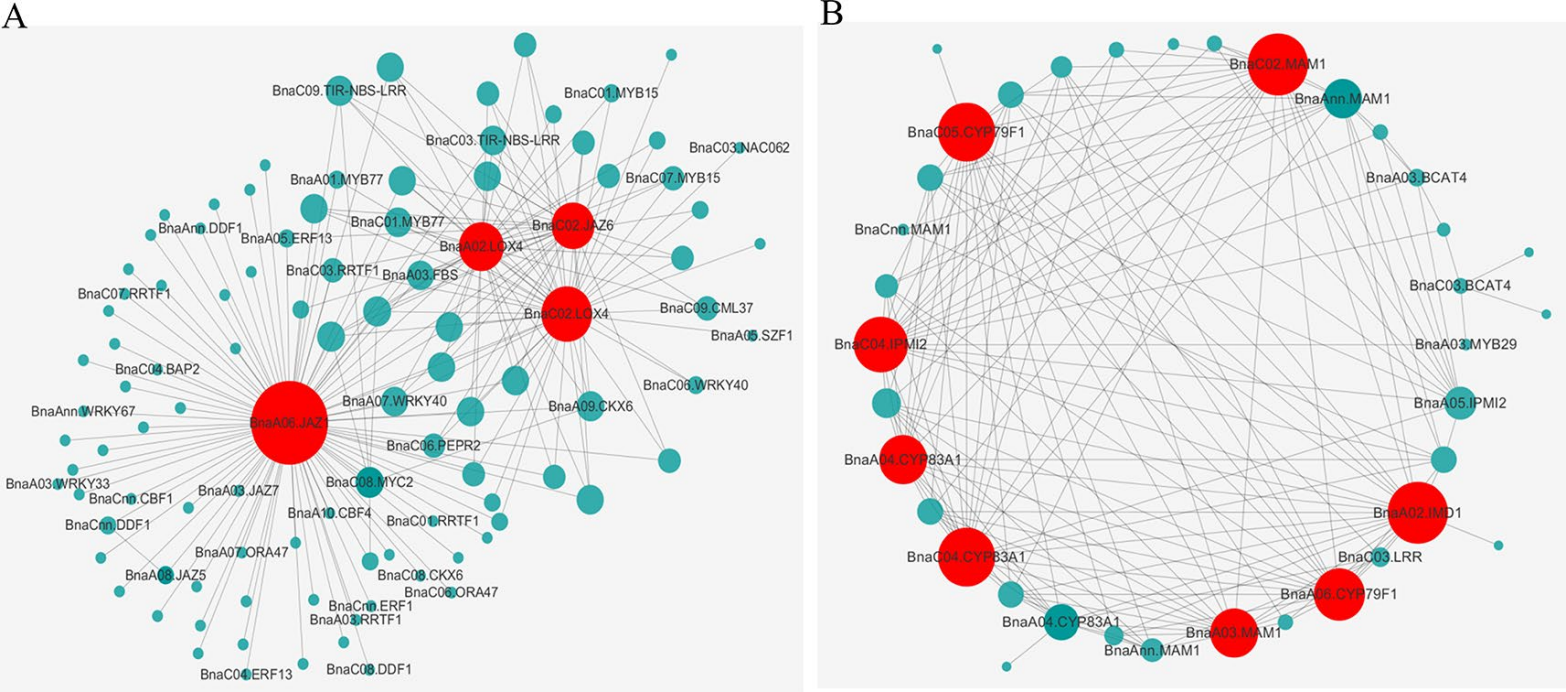
Interaction network of the identified hub genes. The gene co-expression network of “blue” module **(A)** and “purple” module **(B)** revealed the hub genes colored by red.

Furthermore, we performed KEGG enrichment analysis of the above eight modules. The genes of three modules were enriched in six pathways significantly, which were marked with the bold font in the **Table 2**. It was worth mentioning in particularly that the genes of blue (414 RDEGs) and purple (43 RDEGs) modules could be involved in fatty acid metabolism, plant hormone signal transduction, and GLS biosynthesis, which were accordance with the KEGG analysis of all RDEGs (**Table 2**). Subsequently, the interaction network of fatty acid metabolism, plant hormone signal transduction, GLS biosynthesis genes in blue module, and purple module were constructed and visualization using Cytoscape3.6.1. The network showed the lipoxygenase (*LOX4*) in fatty acid metabolism, and jasmonate-zim-domain protein (*JAZ1* and *JAZ6*) in plant hormone signal transduction played pivotal role in the CR (**Figure 5A**). At the same time, isopropylmalate isomerase 2 (*IPMI2*), isopropylmalate dehydrogenase 1 (*IMD1*), methylthioalkylmalate synthase -in pathway of GLS biosynthesis also played an important role in resistance to *P. brassicae* (**Figure 5B**). In total, there were 12 genes were highlighted after WGCNA and interaction network analyses, which were considered to be the hub genes for CR in the R-line (**Figure 5**).

**Table 2:** Kyoto Encyclopedia of Genes and Genomes enrichment analysis of modules associated with R- or S-line significantly in weighted gene co-expression network analysis.

Module	Pathway	Description	p_value	Associated sample
Purple	**ath00966**	**Glucosinolate biosynthesis**	**7.92E−09**	R_12h
	**ath01210**	**2-Oxocarboxylic acid metabolism**	**8.52E−07**	
	**ath00290**	**Valine, leucine, and isoleucine biosynthesis**	**2.69E−04**	
Salmon	ath00950	Isoquinoline alkaloid biosynthesis	1.40E−02	R_12h
	ath00960	Tropane, piperidine, and pyridine Alkaloid biosynthesis	2.05E−02	
	ath00350	Tyrosine metabolism	2.45E−02	
	ath00410	Beta-alanine metabolism	2.62E−02	
	ath00360	Phenylalanine metabolism	2.94E−02	
	ath00260	Glycine, serine, and threonine metabolism	4.71E−02	
	ath00630	Glyoxylate and dicarboxylate metabolism	4.87E−02	
Green	**ath00010**	**Glycolysis/gluconeogenesis**	**9.98E−03**	S_12h
	ath00380	Tryptophan metabolism	1.54E−02	
	ath00620	Pyruvate metabolism	4.94E−02	
Magenta	ath00940	Phenylpropanoid biosynthesis	1.51E−02	S_24h
Pink	ath04712	Circadian rhythm—plant	1.53E−02	R_60h
	ath03008	Ribosome biogenesis in eukaryotes	3.38E−02	
Yellow	ath04016	MAPK signaling pathway—plant	2.33E−02	S_60h
	ath00760	Nicotinate and nicotinamide metabolism	3.47E−02	
Blue	**ath00591**	**Linoleic acid metabolism**	**6.55E−04**	R_96h
	**ath04075**	**Plant hormone signal transduction**	**7.48E−03**	
	ath00592	Alpha-linolenic acid metabolism	1.33E−02	
	ath00920	Sulfur metabolism	1.59E−02	
	ath00400	Phenylalanine, tyrosine, and tryptophan biosynthesis	2.48E−02	

Bold font indicates the genes of three modules enriched in six pathways significantly.

### The Single-Nucleotide Polymorphism and Haplotype Analysis of Pivotal Genes

**Figure 6:**
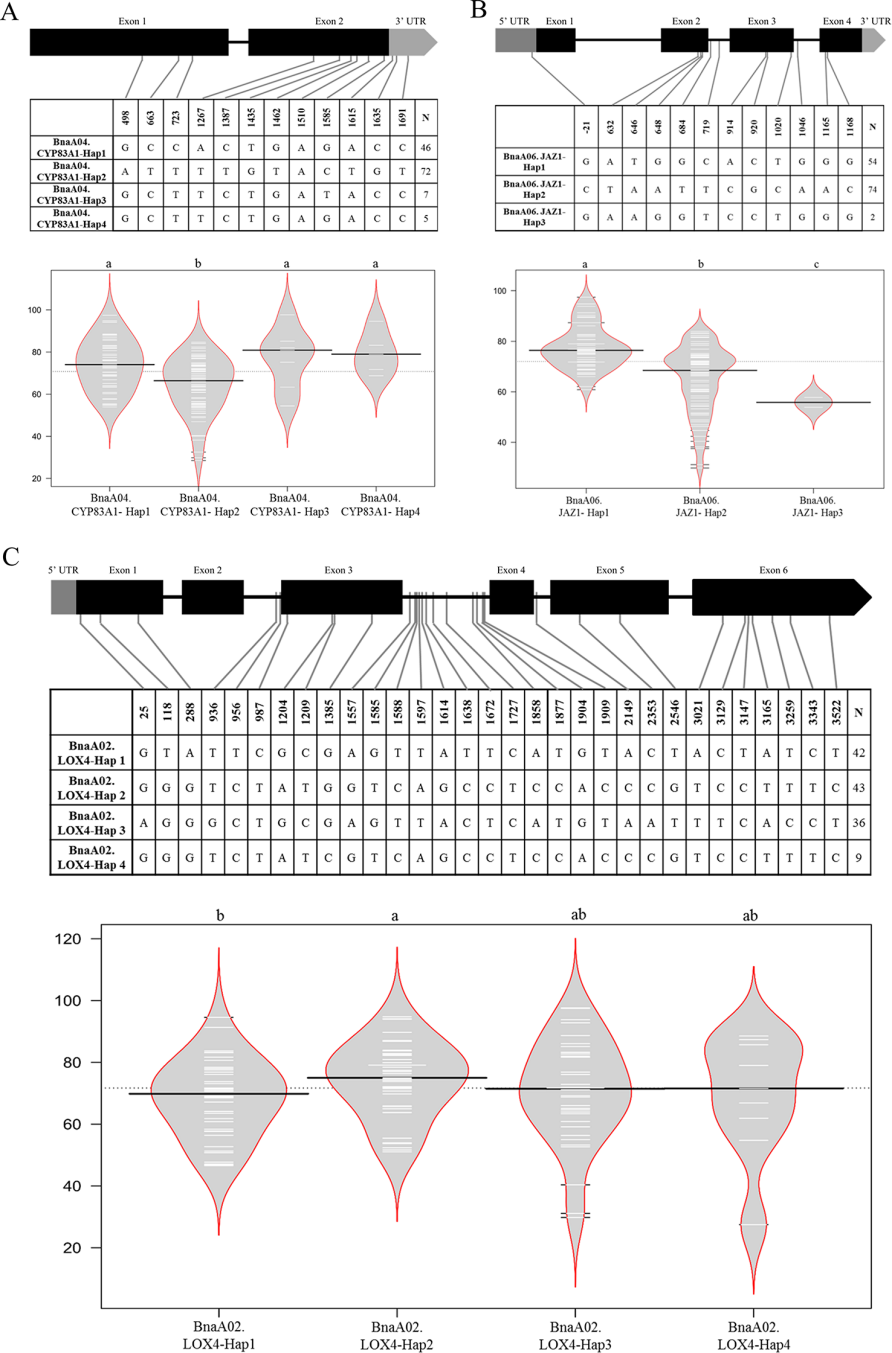
Haplotype analyses of *BnaA04.CYP83A1* **(A)**, *BnaA06.JAZ1* **(B)**, and *BnaA02.LOX4* **(C)**. The exon, and UTR in the gene structure were displayed with black, and gray box, respectively. The single nucleotide polymorphism (SNP) positions were connected to the haplotype table by lines, and the number of accessions carrying each haplotype was indicated in the columns of right. The difference significance analysis among haplotypes was displayed by the violin plot, in which the black and white horizontal lines represented the medians and individual data points, respectively.

Some key genes were obtained through transcriptome analysis, indicating that the changes in the expression levels of these important genes might result in the differences in CR. To better and further understand the function or variation of these hub genes in rapeseed, haplotype analysis was performed on part of hub genes. The gene of *BnaA04.CYP83A1* involved in GLS biosynthesis had two exons, one intron, and 3’ untranslated region (UTR). PCR products were sequenced for *BnaA04.CYP83A1* (1,827 bp) from a population containing 130 accessions. Twelve SNPs were detected in the transcriptional region. Four, one, and two SNPs were in the exon 1, exon 2, and 3’ UTR, respectively. Haplotype analysis of *BnaA04.CYP83A1* showed that four haplotypes were constructed. Hap1 and Hap2 were the prevalent haplotypes, represented by 46 and 72 accessions severally, while Hap3 and Hap4 were rare types represented only by 7 and 5 accessions. Combined with the phenotype of artificial inoculation identification, it was found that the DI of Hap2 (64.18) was significantly lower than that of the other three haplotypes among which there was no significantly difference (**Figure 6A**). The results indicated that Hap2 was the favorable haplotypes of *BnA04.CYP83A1* for CR. Similarly, the gene of *BnA06.JAZ1*, involved in the pathway of plant hormone signal transduction, had four exons, three introns, 5’ UTR, and 3’UTR. The entire *BnaA06.JAZ1* (1,547 bp) was also sequenced in the 130 accessions. One, three, three, and two SNPs were detected in the 5’UTR, exon 2, exon 3, and exon 4, respectively. Haplotype analysis of *BnaA06.JAZ1* showed that three haplotypes were constructed. Hap1 and Hap2 were the prevalent haplotypes, represented by 54 and 74 accessions severally, while Hap3 was rare type represented only by 2 accessions. Phenotype difference of any two haplotypes was significantly and the Hap2 was the favorable haplotype of *BnaA06.JAZ1* for CR (**Figure 6B**). The gene of *BnaA02.LOX4* participated in fatty acid metabolism had six exons, five introns, and 5’ UTR. PCR products were sequenced for *BnaA02.LOX4* (3,757 bp) in the 130 accessions. Three, four, two, and seven SNPs were in the exon 1, exon 3, exon 5, and exon 6, respectively. Haplotype analysis of *BnaA02.LOX4* showed that four haplotypes were constructed. Hap1, Hap2, and Hap3 were the prevalent haplotypes, represented by 42, 43, and 36 accessions severally, while Hap4 was rare type represented only by 9 accessions. Combined with the phenotype data of artificial inoculation identification, it was found that the DI of Hap1 (68.12) was significantly lower than that of Hap2 (73.92, **Figure 6C**), which indicated that Hap1 was the favorable haplotypes of *BnaA02.LOX4* for CR.

To better understand how the mutations were impact on the protein transcriptions or the functions (whether the mutations in the motifs or not), the online software (https://www.genome.jp/tools/motif/ and https://prosite.expasy.org/scanprosite/) were used to predict motifs of above three genes. The results showed that, for BnaA04.CYP83A1, only the SNP (Pos_1510) caused missense mutation that from the isoleucine to methionine, which was not in the motif of this gene (CYTOCHROME_P450), though; for BnA06.JAZ1, there were four missense mutations (Pos_646, 648, 914, and 920), also not in the motifs of the gene (TIFY and CCT_2); for BnaA02.LOX4, there were five missense mutations (Pos_25, 118, 1204, 1385, and 2546), also not in the motifs of the gene (LIPOXYGENASE_1 and LIPOXYGENASE_2).

## Discussion

Many studies focused on the middle or late phase/stage of clubroot course in *Arabidopsis*, *B. rapa* (Chinese cabbage), *Brassica oleracea*, and *B. napus* on the level of transcriptomics, proteomics, or others (Zhang et al., 2016; Hao et al., 2017; Irani et al., 2018; Ji et al., 2018; Prerostova et al., 2018; Su et al., 2018; Peng et al., 2019), while few studies were aimed at the early infection. In recent years, some researchers put forward that the early infection also played an important role. Both *Arabidopsis* and Chinese cabbage were carried out the study on the mechanisms of early infection (Chen et al., 2015; Zhao et al., 2017), which showed that part of pathways or proteins identified in the middle or late phase also could be detected in the early infection stage. In this study, we investigated the response of *B. napus* accessions with different resistance levels at the early stage of *P. brassicae* infection, which could provide more information about the mechanisms of early infection of *Brassica* crops to *P. brassicae*. It speculated that resistant genotype could sense pathogen invasion earlier because that more DEGs were detected in R-line and the 12 h/96 h after infection might be the key time points for the R-line. The dynamic change of RDEGs number showed that the number at 12 h after inoculation was the least. We considered that most resistance reaction at this point in R-line might belong to the basal reaction, which could occur in S-line in spite that it would occur later. In addition, the number of RDEGs reached the maximum at 96 h after inoculation, and there was a growing trend over time. It was conjecture that the main reason for this change was that many specific responding of R-line revealed gradually in this process. Therefore, it illustrated that the sampling time point in this study was desirable.

In this study, the concept of RDEG was introduced. Compared with simple analysis of DEGs in R- and S-line, it was more convinced and targeted to discover the key genes that lead to the difference between R- and S- line. It could filter some genes belonging to basic resistant pathways. The expressional level of these genes had no difference between R- and S-line. Eight metabolic pathways were identified involved in the regulation of CR by KEGG enrichment analysis of RDEGs. Among that, two pathways, GLS biosynthesis and plant hormone signal transduction, have been repeatedly reported to be involved in the regulation of CR (Ludwig-Muller, 2009), which further proved the referential value of the information obtained in this study. It was worth mentioning that the pathway of tryptophan metabolism was considered synergy with above two pathways, because that it was a precursor of auxin and various secondary metabolites, such as camalexin and GLS. A new study indicated that suppression of tryptophan synthase could activate cotton immunity by triggering cell death *via* promoting SA synthesis (Miao et al., 2019). While, few study about another four pathways of fatty acid metabolism, pyridine alkaloid biosynthesis, carotenoid biosynthesis, and sulfur metabolism related to CR was reported. For all this, the four pathways were reported involved in other disease resistance reaction, which also provided some new ideas for study on CR mechanisms. Alkaloids, as an important natural phytoalexin, were accumulated in large amount when plants were stimulated by adverse environment. The relationship between alkaloids and disease resistance was mainly manifested as inhibition of spore germination and mycelial growth of pathogenic fungi, or inhibition and inactivation of enzymes or toxins produced by pathogens. Some plant hormones had inhibitory effects on the synthesis of alkaloids. For example, auxin negatively regulated the synthesis of alkaloids (Kutchan, 1995). Plant immune response was a very complex biological process, there were abundant genes participated in this process. Some key pathways, like GLS biosynthesis and plant hormone signal transduction, were considered as the key pathways in the plant immune response process. Our data also reflected the importance of GLS biosynthesis and plant hormone signal transduction on the CR resistance in rapeseed (**Table 2**, **Figures 4** and **5**). On the other hand, some pathways were indirect involved or influenced the plant immune, like cell wall, lipid metabolism, glycolysis/gluconeogenesis, which might affect the energy flux from pathway to pathway or as the physical barrier to impact the plant disease resistance response. Also, our RNA-Seq data identified the indirect involvement pathways on the CR resistance in rapeseed, like linoleic acid metabolism, sulfate transport, and gluconeogenesis (**Table 2**). It was indicated that these pathways also very important in the plant immune response, despite the effect was indirect. It was also verified that transcriptome was a powerful method for understanding the complex biological questions.

Fatty acids were also involved in the regulation of plant responses to various biotic and abiotic stresses, primary metabolites of which played an important role in signal transduction of plant disease resistance. Oleic and linoleic acid can induce the activation of nicotinamide adenine dinucleoside phosphate oxidase mediated by protein kinase C, thus inducing the production of plant reactive oxygen species involving the plant disease resistance (Cury-Boaventura and Curi, 2005). JA was an important plant hormone derived from fatty acid metabolism. Therefore, it was not surprised that these two pathways were detected in the network of blue module, simultaneously. Fatty acid desaturation was an important part of plant defense reaction and two genes (*LOXs*) encoding lipoxygenase, which catalyzed the oxygenation of fatty acids, were screened out in this study. In the meanwhile, two genes (*JAZs*) encoding jasmonate-zim-domain protein, which involved in the JA signaling pathway were also screened out. JA and its derivatives played an important role in mediating plant resistance to various kinds of biological stress, as well as in the process of vegetative reproduction, cell cycle regulation. In addition, some studies suggested that *JAZ1* maybe connect the auxin and JA signaling pathways. JA was also believed to mediate the anabolism of alkaloids. The results presented a complex metabolic network formed by the interaction of multiple metabolic pathways, which could provide more information to explore the regulation of CR. Although Chen et al. (2015) also identified the *LOXs* and *JAZs* involved in the regulation of *B. rapa* against to *P. brassicae*. However, the results showed that *JAZs* were down-regulated in CR BJN3-2, and it was concluded that the SA signaling pathway, not the JA/ET played a crucial role in resistance of *B. rapa* against to *P. brassicae*. On the contrary, both our study and Zhang et al. (2016) showed that *JAZs* were up-regulated in R-line, inhibiting the JA signaling pathway, to improve the CR. It revealed the importance of JA signaling pathway to the CR. Numerous studies had shown that CR in *B. rapa* was considered as a quality trait, while CR in *B. oleracea* had been considered as a quantitative trait (Piao et al., 2009; Lee et al., 2016). It was a pity that the resistance mechanisms of *B. napus* to *P. brassicae* was still unknown. This study could further reveal the genetic mechanisms of CR in *B. napus*.

GLS, as a kind of secondary metabolite widely existing in cruciferous plants, were broadly existed in *Brassica* species. The degradation products of GLS had widely biological functions, which could not only regulate auxin metabolism, but also participate in plant defense reaction, preventing and controlling plant diseases. In general, the biosynthesis of GLS included three stages: extension of precursor amino side chain, formation of GLS core structure, and modification of side chain groups (Grubb and Abel, 2006). According to the different side chains, the GLS could be divided into aliphatic, aromatic, and indole. It had been proved that different group GLS and their corresponding isothiocyanates were only resistant to specific pathogen (Tierens et al., 2001). Up to now, most studies focused on indole-GLS, while information on aliphatic and aromatic-GLS was limited. Aliphatic-GLS was considered playing the role of defense by releasing toxic thiocyanate and isothiocyanates, while indole-GLS precursor was regarded as the synthesis of auxin, associated with the root of forming large size. It was supposed that indole-GLS may directly or indirectly promote clubroot incidence degree (Ludwig-Muller, 2009). At present, the relevant major synthesis and regulation genes in biosynthesis of GLS have been verified, which were determined by *MAM*, *CYP79*/*CYP83*, *AOP*, and other synthetic gene families (Hull et al., 2000; Hansen et al., 2001; Mikkelsen et al., 2002; Hirai, 2008). The TFs of *MYB* gene family played the role of regulating the above genes (Yan and Chen, 2007; Gigolashvili et al., 2009). The gene, *CYP83A1*, which involved in the formulation of GLS core structure, was screened as a candidate gene by combining functional enrichment analysis with co-expression network analysis in this study. Meanwhile, the contribution of *CYP83A1* to CR was verified in a population by correlation analysis of CR phenotypes with the haplotypes of *CYP83A1* in rapeseed. Studies showed that *CYP83A1* had a high affinity for aliphatic acetaldoxime compared with its homologous gene *CYP83B1*, which had a high affinity for indole acetaldoxime (Bak and Feyereisen, 2001). The results of this study indicated that aliphatic-GLS also played an important role in the regulation of CR, which could extend the understanding on the contribution of different group of GLS to CR in *Brassica*.

In addition, WGCNA analysis also revealed genes interacting with above key genes, especially some important TFs and *R* genes, which were worthy of attention in the following study. Several TFs (*WRKY33*, *WRKY40*, *WRKY67*, *MYB15*, *MYB77*, and *MYC2*) were identified in the blue module network, which were reported to involve in defense to biotic stress (Lorenzo et al., 2004; Pandey et al., 2010; Birkenbihl et al., 2012; Chezem et al., 2017). In addition, two R genes (TIR-NBS-LRR) were identified from the network of blue related to the fatty acid metabolism and plant hormone signal transduction. The expressional data showed that both R genes were induced express after 72 h infected by *P. brassicae*. And compared with the S-line, both R genes were up-regulated in the R-line. WGCNA analysis could provide valuable information for the establishment of regulatory network of CR in rapeseed.

Through the RNA-Seq analysis at the early stage of the infection the *P. brassicae*, a total of 12 hub genes related to club root resistance were obtained by WGCNA and interaction network analyses. The information of these genes is powerful for CR improvement breeding in *Brassica*. Haplotype and mutations details resulted by SNPs analyses were performed in this study (**Figure 6**). It could be concluded that the natural variations of target genes could affect the CR, although these variations were not located on the motif of the genes. Even so, the mutations might cause the protein substrate binding ability or enzyme activity had slightly changes, thus results in the phenotype differences. The results appeared to be particularly important for understanding the mechanisms on CR resistance in *B. napus*.

## Data Availability Statement

The raw transcriptome reads have been deposited into NCBI Short Read Archive (SRA) under accession number PRJNA564005.

## Author Contributions

XW conceived the study. LL and XW designed the experiments. LL organized the implementation and analyzed the data of experiment. YL and HL participated in the RT-PCR and phenotype identification. LL wrote the paper. All the authors have read and approved the publication of the manuscript.

## Funding

The National Key Program for Research and Development (2016YFD0100202) and The Germplasm Resources Protection Project in China (2019NWB040) supported this work.

## Conflict of Interest

The authors declare that the research was conducted in the absence of any commercial or financial relationships that could be construed as a potential conflict of interest.
