# Supplementary material for: Comparative Transcriptome Analysis Reveals Key Pathways and Hub Genes in Rapeseed During the Early Stage of Plasmodiophora brassicae Infection
Source: Front Genet. 2020 Jan 17;10:1275. doi: 10.3389/fgene.2019.01275 (PMC6978740; doi:10.3389/fgene.2019.01275)
Supplement: Supplementary file 1 [file DataSheet_1.docx]

**Comparative transcriptome analysis reveals key pathways and hub genes in rapeseed** **during the early stage of *Plasmodiophora brassicae* infection**

***Lixia Li, Ying Long, Hao Li and Xiaoming Wu****

Key Laboratory of Biology and Genetic Improvement of Oil Crops, Ministry of Agriculture and Rural Affairs, Oil Crop Research Institute, Chinese Academy of Agricultural Sciences, Hubei, China

***Correspondence:**

Dr. Wu Xiaoming

wuxm@oilcrops.cn

Tel: +86 27 86812906

**RUNNING TITLE:**

RNA-seq for rapeseed resists clubroot

| **Table S1 Gene-speciﬁc primers sequences of RT-PCR** | | |
| --- | --- | --- |
| **Gene ID** | **Forward** | **Reverse** |
| *BnaA02g02020D* | GATAGAATTGCTCGTGTTGCAT | GTCTGGATACTCAGAGGCTAAC |
| *BnaA02g30740D* | TCAAGTAAACGGAAAGCCTACT | CCTCAGGAGTTGTGGTATAGTG |
| *BnaA02g34540D* | CCGGTTAGCAGAATAGTTTTCG | CAAAACGTCCGAGCTTATACAC |
| *BnaA02g35250D* | AACCGACCAATGAGAATGTACT | TCTCATCCTTTCTTGAGCAGTT |
| *BnaA03g20310D* | GGTCCTAACCTTAGCTGCTAAA | GGGAACACTAAGACCCAACATA |
| *BnaA03g52720D* | TGTTGAAGCAGTTAACATGTGG | CCACTCGCCTACAATATTACCT |
| *BnaA04g03320D* | GAGAGGTTGTCATTGGCATTTT | TCGTGTAAGCACATTCCACTAT |
| *BnaA04g21450D* | GAACAAGTGTGATTTCTTCGCT | TGTCCGAATCTTGACTTCTGAA |
| *BnaA04g22000D* | TGAAAGCAGAGATTGAGGAAGT | CCCGACAAAAGCACTGATATTT |
| *BnaA04g24160D* | ATGTTTCGAGAGACAAGACACT | CATCAAGAGGTCAATCATGCTG |
| *BnaA05g03210D* | CTCCTTTCAAAGCCTTCACTTC | TATGAGCAGCAGTATCCGTATG |
| *BnaA06g23250D* | GCTGCTAAAACTCTCAACACAA | TCCTTAGGAACGATGTGTCAAA |
| *BnaA07g31260D* | CCTTACGTCGAGATAGACTTCG | TTCGGGTCTCTCCAAATGTAAA |
| *BnaA10g09270D* | CAACTGTCAGAAGCTGAAACTC | GTTTGGTATGGCTTGAGCATAG |
| *BnaA10g22140D* | TACTAGTGGTGGAGGAATACGA | AACTCTCCATCCTCTTTTCCTG |
| *BnaC02g19140D* | GGAGCTTCTAAGATTAGCAAAAGC | GGTAGGTGTTTTATCATCCACC |
| *BnaC02g42920D* | GAGTCGACTTAGACGAAGGTAG | GTCTACGCAAGCCTAATAAACG |
| *BnaC02g45730D* | TGTGTTATGATGCTGTCTGGAT | GGAAAGTTGTCGCATTTCAGAA |
| *BnaC03g18820D* | AATCATGTTTCCATTAGCCACG | CATTACCACCGTTGAAAACCAA |
| *BnaC03g27470D* | CCCTGGCTTCATACGTGTATAT | CCTTCTCGTTCATGGTTGGATA |
| *BnaC04g29320D* | CTCACGGCTTATATGATGGAGT | ATCGATCATGCTCTCAGTTTCT |
| *BnaC04g46230D* | ACCAGAAGCTTCAGAGTTAGTC | GCATCGATATAACGAATGGCTC |
| *BnaC04g52990D* | CCGACGTTTTGTTACACAAGAT | ATTAGCGATCGTTTGGAAACTG |
| *BnaC05g12520D* | TACGGTGAACAGTTCATGAAGA | GTTGTCCGCTTCAATAGTTCTC |
| *BnaC05g48680D* | CTTTCATGACTTTGGTGTCTCG | CGCCGAGAGATTTTTCATTCTT |
| *BnaC06g24340D* | ATCTCTCACCCACTCTTTCAAG | CACTGTTAAGCATCCACAACAA |
| *BnaC07g44430D* | TCAACGAGAAGAACGACTACTT | CACACAACTTGCGTATAGTGAC |
| *BnaC09g50780D* | TCTTAGGGACAAAAGAGTTGCA | CAAAGACCACATCAAATGCTGT |
| *BnaCnng75920D* | GCTAAGCTTACTGAGGTCAGAA | TGTCGATGATGATCTTACGTCC |
| *Bna ACTIN7* | CTGGAATTGCTGACCGTATGAG | ATCTGTTGGAAAGTGCTGAGGG |

| **Table S2 Primers sequences of hub genes** | |  |
| --- | --- | --- |
|  | **Forward** | **Reverse** |
| *BnaA02.LOX4* | AGAAAATGTACGACTTGTTTTA | TTAATTAATAAATTATAACAAG |
| *BnaA04.CYP83A1* | GGACGTTGGGTAGGTGAATGCT | ATAGTACACTACAGGAACGTGT |
| *BnaA06.JAZ1* | AAAAACTGAAGAAGAAGAAGAT | TATAATTAAGAACCATGGACGC |

| **Table S3 Sequencing and mapping statistics for the 32 transcriptomes data** | | | | | | | |
| --- | --- | --- | --- | --- | --- | --- | --- |
| **Sample** | **Total raw_reads** | **Total clean_reads** | **Clean_bases (G)** | **Q30 (%)** | **GC content (%)** | **Total_mapped percentage (%)** | **Unique_mapped percentage (%)** |
| R1_1 | 52352404 | 51104494 | 7.67 | 94.42 | 46.71 | 89.03 | 85.02 |
| R1_2 | 58156738 | 56990858 | 8.55 | 92.34 | 46.55 | 88.35 | 84.42 |
| R1_3 | 57735450 | 56269776 | 8.44 | 92.38 | 46.67 | 88.28 | 84.41 |
| R1_C | 61148308 | 59268258 | 8.89 | 92.45 | 46.77 | 88.15 | 84.23 |
| R2_1 | 61749274 | 60235720 | 9.04 | 92.54 | 46.48 | 87.97 | 84.01 |
| R2_2 | 62896128 | 61182536 | 9.18 | 91.52 | 46.51 | 88.11 | 84.23 |
| R2_3 | 58729910 | 57319260 | 8.6 | 92.89 | 46.51 | 88.46 | 84.49 |
| R2_C | 55351436 | 54089572 | 8.11 | 92.75 | 46.52 | 88.46 | 84.52 |
| R3_1 | 55428944 | 54306366 | 8.15 | 93.05 | 46.4 | 89.27 | 85.44 |
| R3_2 | 54627318 | 53737592 | 8.06 | 91.65 | 46.43 | 88.53 | 84.82 |
| R3_3 | 56250724 | 55256972 | 8.29 | 92.47 | 46.56 | 88.63 | 84.66 |
| R3_C | 56547992 | 55760872 | 8.36 | 91.25 | 46.58 | 88.42 | 84.76 |
| R4_1 | 56615210 | 55653404 | 8.35 | 92.23 | 46.58 | 88.46 | 84.73 |
| R4_2 | 62841364 | 61583438 | 9.24 | 92.31 | 46.43 | 88.16 | 84.41 |
| R4_3 | 58746700 | 57907958 | 8.69 | 92.62 | 46.56 | 87.24 | 83.57 |
| R4_C | 68288108 | 67172902 | 10.08 | 94.1 | 46.68 | 89.02 | 85.06 |
| S1_1 | 59933094 | 58857590 | 8.83 | 94.13 | 46.82 | 90.11 | 86.07 |
| S1_2 | 55205506 | 54425512 | 8.16 | 94.37 | 46.69 | 90.21 | 86.24 |
| S1_3 | 55905884 | 55107718 | 8.27 | 93.21 | 46.66 | 89.62 | 85.82 |
| S1_C | 46839588 | 46131370 | 6.92 | 88.75 | 46.93 | 88.01 | 84.19 |
| S2_1 | 49101116 | 48325866 | 7.25 | 92.69 | 46.6 | 87.96 | 84.03 |
| S2_2 | 54217540 | 53214384 | 7.98 | 93.09 | 46.68 | 89.06 | 85.05 |
| S2_3 | 51663072 | 50880012 | 7.63 | 93.19 | 46.66 | 88.89 | 84.98 |
| S2_C | 51978778 | 51123310 | 7.67 | 92.89 | 46.7 | 89.31 | 85.42 |
| S3_3 | 49796306 | 47518234 | 7.13 | 92.81 | 46.56 | 90.16 | 86.27 |
| S3_2 | 46952282 | 46056550 | 6.91 | 93.07 | 46.59 | 90.3 | 86.42 |
| S3_1 | 48624418 | 49046254 | 7.36 | 92.55 | 46.66 | 89.43 | 85.69 |
| S3_C | 46448382 | 45734808 | 6.86 | 92.42 | 46.69 | 89.33 | 85.62 |
| S4_1 | 54920762 | 54181064 | 8.13 | 93.17 | 46.67 | 90.08 | 86.32 |
| S4_2 | 50260538 | 49355958 | 7.4 | 92.99 | 46.78 | 88.97 | 85.2 |
| S4_3 | 61160492 | 60273378 | 9.04 | 93.09 | 46.68 | 89.72 | 85.96 |
| S4_C | 50589062 | 49976216 | 7.5 | 93.42 | 46.67 | 89.6 | 85.87 |

**FIGURE S1 Pearson correlation coefficients (PCC) of three biological replicates**

**FIGURE S2 Venn diagram of DEGs in four sampling time points in R- (a) and S-line (b)**

**FIGURE S3 Correlation of 29 RDEGs selected randomly ratios obtained by RNA-seq and RT-PCR.** The y-axis means the log2 fold change of RNA-seq and the x-axis means that of RT-PCR. The fold change calculated by R/S. All RT-PCR data were collected from three technical replicates for each sampling time point. (A) 12 hpi; (B) 24 hpi; (C) 60 hpi; (D) 96hpi.

**TABLE S1 Gene-speciﬁc primers sequence of RT-PCR**

**TABLE S2 Primer sequences of hub genes**

**TABLE S3 Sequencing and mapping statistics for the 32 transcriptomes data**
