# Supplementary figures and images for: Comparative Transcriptome Analysis Reveals Key Pathways and Hub Genes in Rapeseed During the Early Stage of Plasmodiophora brassicae Infection
Source: Front Genet. 2020 Jan 17;10:1275. doi: 10.3389/fgene.2019.01275 (PMC6978740; doi:10.3389/fgene.2019.01275)

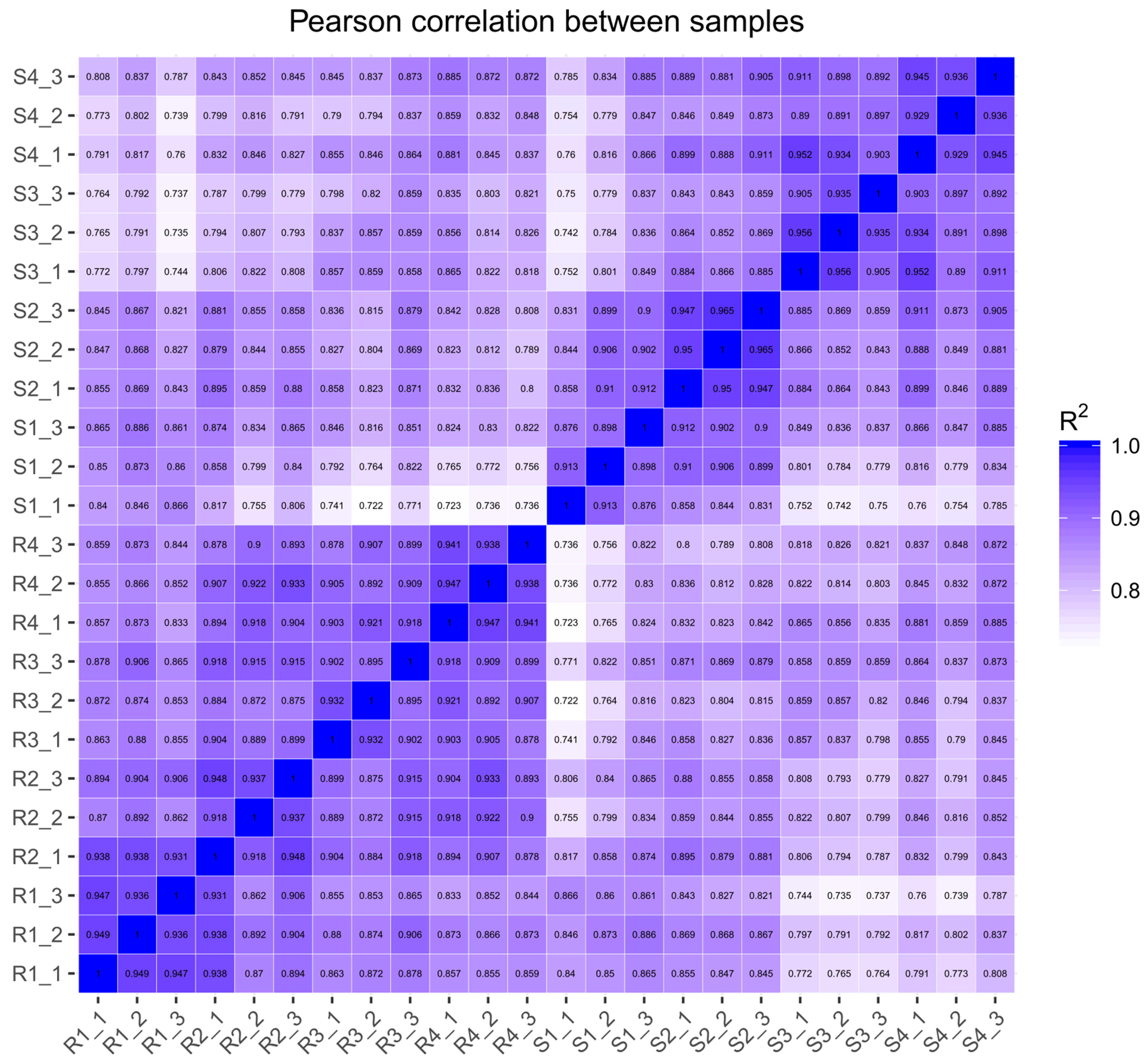

Supplement: Supplementary file 2 [file Image_1.tif]

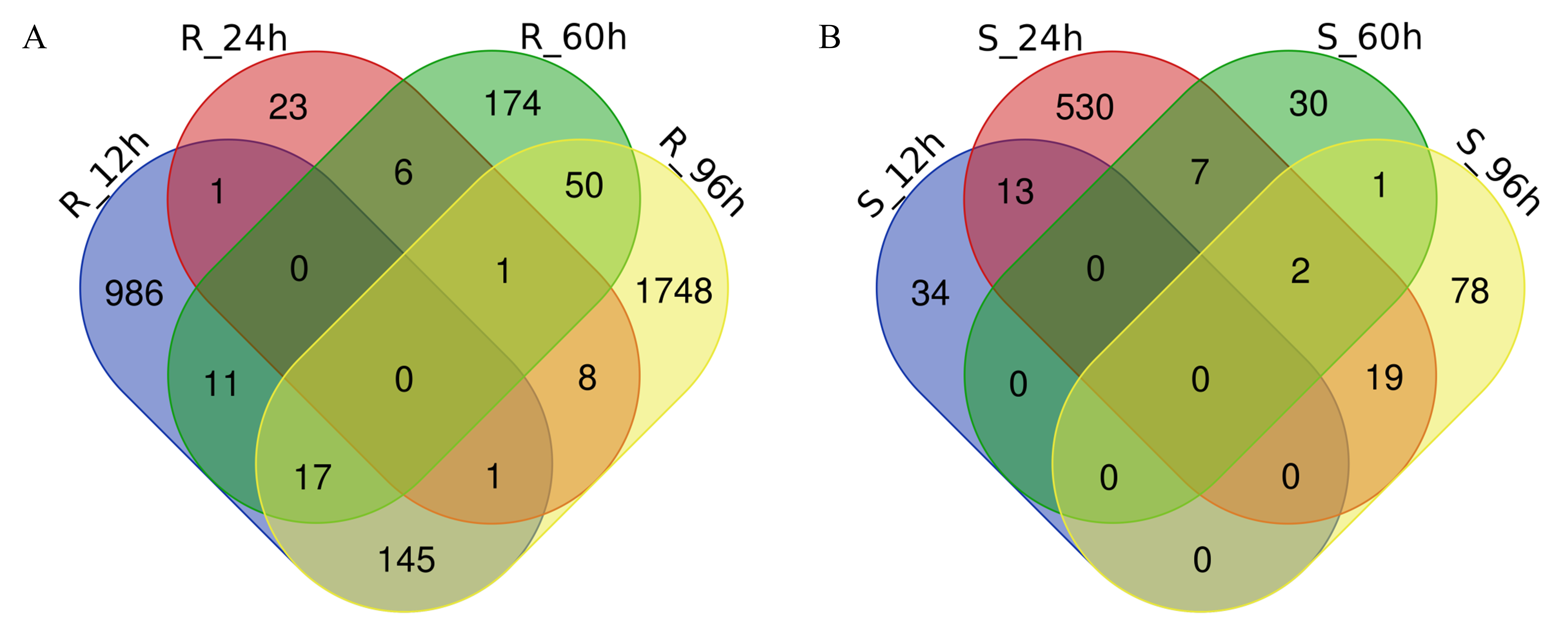

Supplement: Supplementary file 3 [file Image_2.tif]

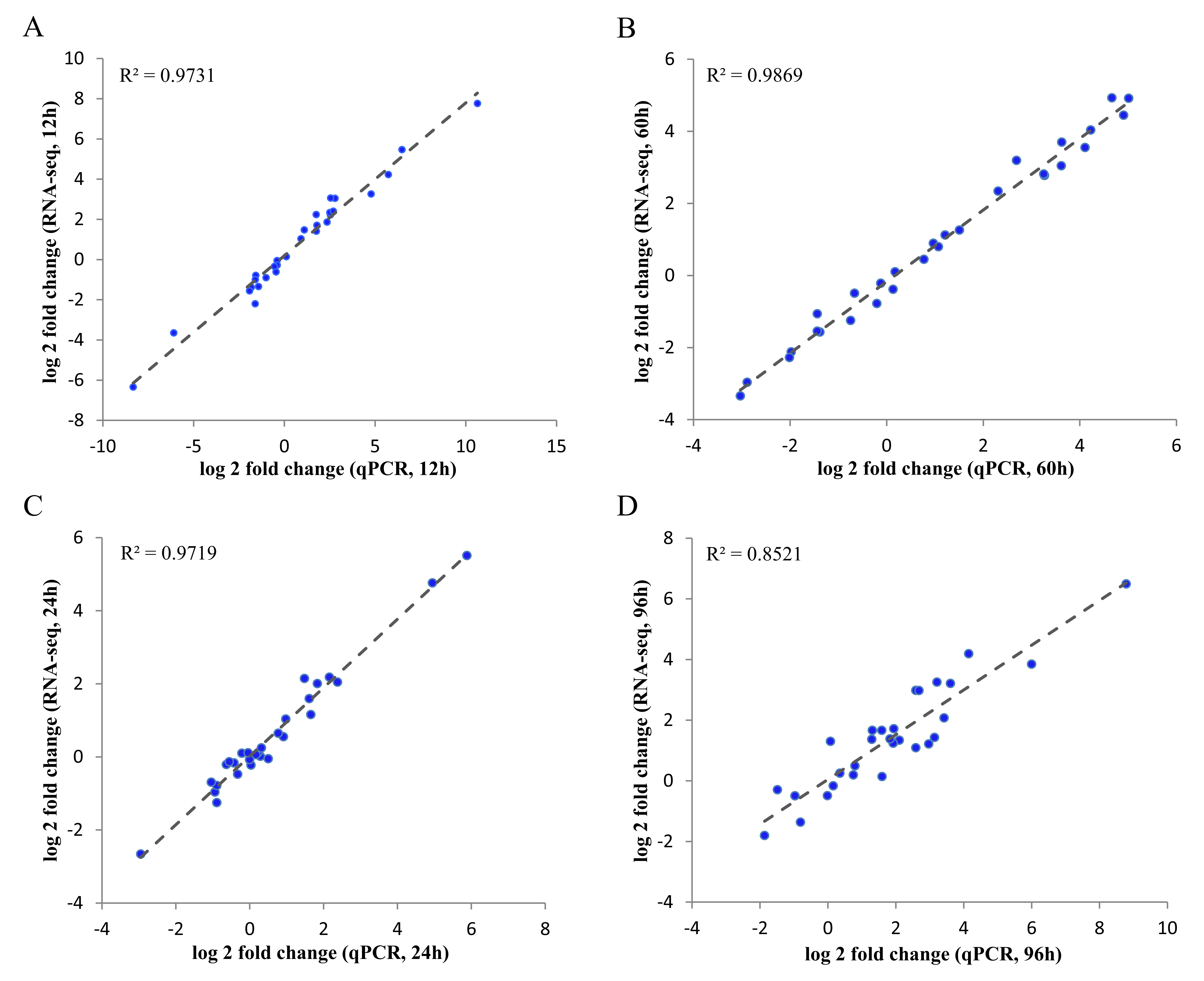

Supplement: Supplementary file 4 [file Image_3.tif]
